# Rituximab Treatment in Hepatitis C Infection: An *In Vitro* Model to Study the Impact of B Cell Depletion on Virus Infectivity

**DOI:** 10.1371/journal.pone.0025789

**Published:** 2011-09-30

**Authors:** Zania Stamataki, Samantha Tilakaratne, David H. Adams, Jane A. McKeating

**Affiliations:** 1 Medical Research Council Centre for Immune Regulation, University of Birmingham, Birmingham, United Kingdom; 2 National Institute for Health Research Biomedical Research Unit and Centre for Liver Research, University of Birmingham, Birmingham, United Kingdom; University of Kansas Medical Center, United States of America

## Abstract

Hepatitis C virus (HCV) infected patients with vasculitis are often treated with the B-cell-depleting anti-CD20 antibody rituximab. Treatment reduces the cryoglobulins that cause vasculitis, yet it also leads to a transient increase in liver enzymes and HCV genomic RNA in the periphery. The mechanism underlying the increased viral load is unclear and both direct and indirect roles have been proposed for B cells in HCV infection. We previously reported that HCV can associate with B cells and can trans-infect hepatocytes. We established an *in vitro* assay to study the effect(s) of rituximab on B cell-associated HCV infectivity. Rituximab-mediated lysis of B cells *in vitro* increases the level of infectious HCV released from B cells. Our results, using a model where virus does not replicate in B cells, recapitulate observations seen in patients and may explain in part the rapid increase in blood HCV RNA observed after rituximab treatment.

## Introduction

HCV infection predominantly affects the liver but extrahepatic manifestations have been reported [1], [2]. Patients who present with symptoms of vasculitis caused by type II cryoglobulinaemia can benefit from treatment with the chimeric monoclonal anti-CD20 antibody rituximab, which depletes B cells in the circulation. Rituximab was first developed to target malignant B cells [3], [4]. Pre-B cells, immature, mature and activated B cells all express CD20 and are susceptible to antibody-dependent lysis [5]. In contrast, hematopoietic progenitor cells, pro-B cells and differentiated antibody-producing plasma cells do not express CD20 and are insensitive to rituximab: the distribution of CD20 allows for a reversible effect and has no influence on high affinity class-switched antibodies [6].

**Figure 1:**
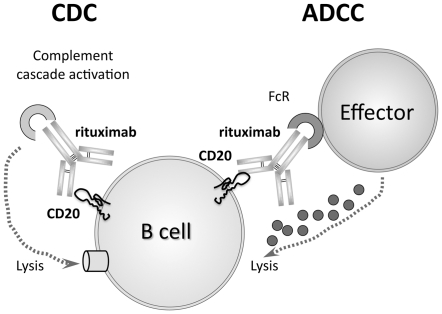
Rituximab mode of action. Rituximab is a monoclonal antibody that recognises the transmembrane receptor CD20 on B cells. Patient treatment with rituximab results in rapid depletion of CD20+ B cells from the circulation, where B cells are deleted predominantly via antibody-mediated and complement-dependent cytotoxicity (ADCC and CDC respectively). NK cells are thought to be the major ADCC-mediating effector cells.

The main modes of rituximab action include antibody-dependent cellular cytotoxicity (ADCC) and complement-dependent cytotoxicity (CDC) (Figure 1), however, apoptosis and phagocytosis have been implicated in B cell depletion [7], [8], [9]. Treatment reduces the level of antibodies that drive cryoglobulin formation and alleviates the clinical symptoms of vasculitis, yet treatment is often associated with a transient increase in liver enzymes and peripheral HCV viral load [10], [11].

It has been challenging to demonstrate that HCV replicates in B cells [12] and recent reports suggest that the JFH-1 strain of HCV that replicates in cell culture (HCVcc) [13], [14], [15] does not productively infect lymphocytes [16], [17]. We recently demonstrated that although B cells do not support HCV replication they can bind HCVcc and trans-infect hepatocytes [17].

Lake-Bakaar and colleagues studied HCV kinetics in infected patients treated with rituximab [10]. The re-appearance of B cells following treatment coincided with a decrease in viral load, suggesting that B cells may serve a protective role. It is not clear why B cell depletion would lead to an increase in peripheral HCV RNA and direct and indirect roles have been proposed for B cells in controlling infection. To address this question, we established an in vitro ADCC model to study the effects of rituximab on B cell associated HCV. We used NK cell degranulation as an indirect measure of cytotoxicity induced by rituximab, and determined the amounts of infectious virus shed by the target B cells. This system revealed that B cells lysed in a rituximab-dependent manner could release a substantial amount of infectious HCV.

## Materials and Methods

### Primary cells, cell lines and viruses

Peripheral blood mononuclear cells (PBMC) were isolated from whole blood by density gradient centrifugation from healthy volunteers who gave informed written consent for participation in the study according to the Declaration of Helsinki. Ethical approval was obtained by the South Birmingham Local Research Ethics Committee (Queen Elizabeth Hospital, Birmingham, UK) and the University Hospital Birmingham Trust. We used the Group I Burkitt's lymphoma B cell line L3055 as a target for the ADCC assay (a kind gift from Prof. J. Gordon, University of Birmingham) and Jurkat T cells as controls (ATCC). Cells were isolated and/or propagated as described [17]. HCVcc JFH-1 was generated and used to infect Huh-7 cells as described [17]. The permissive Huh-7 hepatoma cell line was used to measure infectious HCV.

### ADCC assay

PBMC were cultured in the presence of 100 IU/ml IL-2 for 24 hours to stimulate Natural Killer (NK) cells. Target L3055 B cells were incubated with HCVcc JFH-1 for 2 hours, unbound virus was removed by extensive washing and the cells were then treated with rituximab or control antibody at 10 µg/ml for 30 minutes. Activated PBMC were co-cultured with target cells at 1:10 effector:target (E:T) ratio for 3-7 hours. Rituximab activity was measured by a flow cytometric CD107a NK cell degranulation assay [18]. B cell lysis was confirmed by trypan blue uptake.

### Statistical analysis

Data were presented as means from three or four replicates. Error bars represent standard deviations and comparisons between samples were made using nonparametric tests (Mann-Whitney for unpaired samples and Wilcoxon for paired samples) using Prism 4.0 (GraphPad Software, San Diego, CA).

## Results

**Figure 2:**
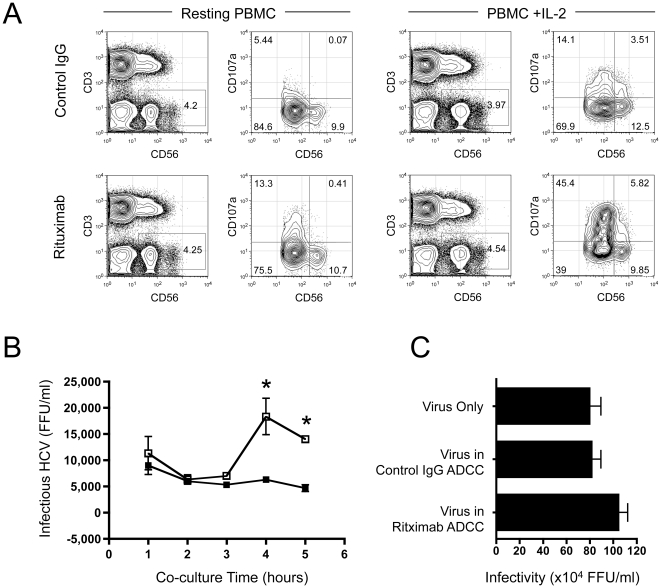
An in vitro model to study the effect(s) of rituximab on B cell-associated HCV. L3055 B cells were exposed to HCVcc JFH-1 for 2 hours, washed to remove non-associated virus and treated with rituximab or control antibody. Resting or IL-2 stimulated PBMC from healthy individuals were co-cultured with the target cells for 4 hours and CD107a content measured in NK cells by flow cytometry. The plots indicate gating on the CD3-CD56+ NK cells, which were further analysed for CD56 expression levels and CD107a marker for degranulation (A). CD56^low^ NK cells are the subpopulation responsible for degranulation following rituximab treatment. The level of infectious virus released by L3055 B cells treated with rituximab (white squares) or control antibody (black squares) was measured by harvesting supernatants from the ADCC assay and assessing infectivity using the permissive Huh-7 hepatoma cell line. Data are presented as focus forming units/ml (FFU/ml) (B). HCVcc JFH-1 infectivity was comparable following incubation in media for 5 hours at 37°C (virus only), or in the presence of B cell ADCC assays in the presence of rituximab or control antibody (C). Error bars represent means from at least 4 replicates ± SD, * p<0.05 compared to control treatment.

To mimic the effects of rituximab on CD20+ B cell depletion by blood cells, we used PBMC from healthy donors as effectors and the Burkitts lymphoma B cell line L3055 as target cells. We previously reported that B cells bound optimal levels of HCV after 2 hours [17], we therefore incubated target B cells with HCVcc JFH-1, washed extensively to remove non-associated virus and treated with rituximab or control antibody at 10 µg/ml. Resting or IL-2 (100 IU/ml) stimulated PBMC were incubated with the target cells at a ratio of 1∶10 for the times indicated in Figure 2. Successful effector activation by rituximab was measured by a flow cytometry-based assay that measures NK cell activation as a factor of CD107a degranulation (Figure 2A). The specificity of rituximab was confirmed using Jurkat T cells as targets, which do not express CD20 (data not shown). As expected, IL-2 pre-treatment of PBMC increased NK cell degranulation and CD107a expression (Figure 2A). Rituximab treatment consistently induced approximately 60% of NK cells to degranulate, independent of PBMC donor variations (data not shown).

We sampled the extracellular media from the ADCC assay for infectious virus released by the lysed target B cells over time (Figure 2B). There was a significant increase in the level of infectious virus after 4 hours of ADCC (p = 0.0442), which coincided with effective B cell lysis. One consequence of the in vitro ADCC assay where target cells are lysed, would be the release of soluble factors that may affect HCV infectivity. It was therefore important to determine the effect(s) of ADCC-mediated soluble factors on HCV infectivity; to this effect, we performed independent ADCC assays in the presence of HCVcc JFH-1 and compared its infectivity to input virus (Figure 2C). Our findings demonstrate that HCV infectivity was not affected by the presence of PBMC/L3055 co-cultures with rituximab or control antibody.

## Discussion

For these experiments, we used HCVcc JFH-1, a genotype 2a strain that replicates in hepatocytes in vitro and in vivo but does not replicate in B cells. Despite the lack of productive infection, our study demonstrated that B cells lysed by effector blood cells could release up to four times more infectious virus following rituximab treatment.

Use of primary B cells from peripheral blood of healthy donors as targets instead of L3055 cells yielded comparable results (data not shown), however this may not reflect the ability of B cells in vivo to capture virus. It is worth noting that L3055 B cells used as rituximab targets, as well as B cells from healthy donors, were not specific for HCV; indeed we previously demonstrated that primary PBMC-derived B cells and L3055 cells bind comparable amounts of HCVcc RNA as measured by quantitative RT-PCR [17]. This was in the order of ∼2,000 RNA copy numbers per 106 B cells (or L3055 B cells), which was relatively low compared to the ∼55,000 RNA copy numbers per 106 permissive Huh-7.5 cells. This study demonstrated virus interaction with B cell-expressed C-type lectins DC-SIGN (dendritic cell–specific ICAM-3–grabbing nonintegrin) and L-SIGN (liver/lymph node–specific ICAM-3–grabbing nonintegrin), and the scavenger receptor class B member I (SR-BI). C-type lectins were expressed in a small B cell subset, whereas SR-BI was present on all B cells. The ability of different B cell subsets to capture HCV remains to be defined.

Our in vitro ADCC assay does not take into account potential virus replication in B cells that may occur in vivo, or the role of B cells in controlling infection via immune protection or indirect clearance mechanisms; despite these limitations, we demonstrated a significant increase in the level of infectious HCV released from B cells in the presence of rituximab which may contribute to the increased viral load observed in patients. In summary, our data support a role for rituximab lysis of B cells and release of infectious HCV as a mechanism to explain the transient increase in viral load observed in treated patients.

## References

[1] Hoofnagle JH (1997). Hepatitis C: the clinical spectrum of disease.. Hepatology.

[2] Galossi A, Guarisco R, Bellis L, Puoti C (2007). Extrahepatic manifestations of chronic HCV infection.. J Gastrointestin Liver Dis.

[3] Maloney DG, Grillo-Lopez AJ, Bodkin DJ, White CA, Liles TM (1997). IDEC-C2B8: results of a phase I multiple-dose trial in patients with relapsed non-Hodgkin's lymphoma.. J Clin Oncol.

[4] McLaughlin P, Grillo-Lopez AJ, Link BK, Levy R, Czuczman MS (1998). Rituximab chimeric anti-CD20 monoclonal antibody therapy for relapsed indolent lymphoma: half of patients respond to a four-dose treatment program.. J Clin Oncol.

[5] Pescovitz MD (2006). Rituximab, an anti-cd20 monoclonal antibody: history and mechanism of action.. Am J Transplant.

[6] Uchida J, Lee Y, Hasegawa M, Liang Y, Bradney A (2004). Mouse CD20 expression and function.. Int Immunol.

[7] Glennie MJ, French RR, Cragg MS, Taylor RP (2007). Mechanisms of killing by anti-CD20 monoclonal antibodies.. Mol Immunol.

[8] Cragg MS, Glennie MJ (2004). Antibody specificity controls in vivo effector mechanisms of anti-CD20 reagents.. Blood.

[9] Cartron G, Watier H, Golay J, Solal-Celigny P (2004). From the bench to the bedside: ways to improve rituximab efficacy.. Blood.

[10] Lake-Bakaar G, Dustin L, McKeating J, Newton K, Freeman V (2007). Hepatitis C virus and alanine aminotransferase kinetics following B-lymphocyte depletion with rituximab: evidence for a significant role of humoral immunity in the control of viremia in chronic HCV liver disease.. Blood.

[11] Sansonno D, De Re V, Lauletta G, Tucci FA, Boiocchi M (2003). Monoclonal antibody treatment of mixed cryoglobulinemia resistant to interferon alpha with an anti-CD20.. Blood.

[12] Stamataki Z (2010). Hepatitis C infection of B lymphocytes: more tools to address pending questions.. Expert Rev Anti Infect Ther.

[13] Lindenbach BD, Evans MJ, Syder AJ, Wolk B, Tellinghuisen TL (2005). Complete replication of hepatitis C virus in cell culture.. Science.

[14] Wakita T, Pietschmann T, Kato T, Date T, Miyamoto M (2005). Production of infectious hepatitis C virus in tissue culture from a cloned viral genome.. Nat Med.

[15] Zhong J, Gastaminza P, Cheng G, Kapadia S, Kato T (2005). Robust hepatitis C virus infection in vitro.. Proc Natl Acad Sci U S A.

[16] Marukian S, Jones CT, Andrus L, Evans MJ, Ritola KD (2008). Cell culture-produced hepatitis C virus does not infect peripheral blood mononuclear cells.. Hepatology.

[17] Stamataki Z, Shannon-Lowe C, Shaw J, Mutimer D, Rickinson AB (2009). Hepatitis C virus association with peripheral blood B lymphocytes potentiates viral infection of liver-derived hepatoma cells.. Blood.

[18] Alter G, Malenfant JM, Altfeld M (2004). CD107a as a functional marker for the identification of natural killer cell activity.. J Immunol Methods.

